# Supplementary material for: Pre- and post-sequencing recommendations for functional annotation of human fecal metagenomes
Source: BMC Bioinformatics. 2020 Feb 24;21:74. doi: 10.1186/s12859-020-3416-y (PMC7041091; doi:10.1186/s12859-020-3416-y)
Supplement: Supplementary file 1 — Additional file 1. This PDF file contains supplementary figures, Figures S1-S5. [file 12859_2020_3416_MOESM1_ESM.pdf]

Supplementary Figures for Treiber ML, et al. “Pre- and post-sequencing recommendations for functional annotation of human fecal metagenomes” BMC Bioinformatics (2020)

Figure S1

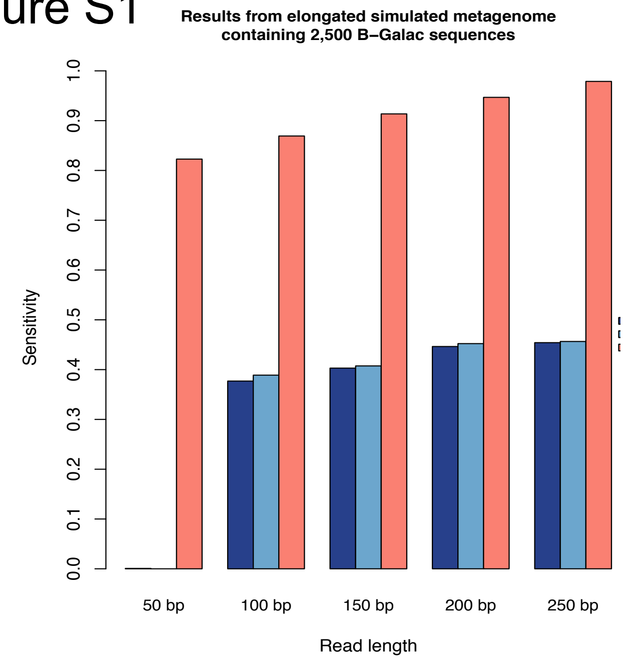

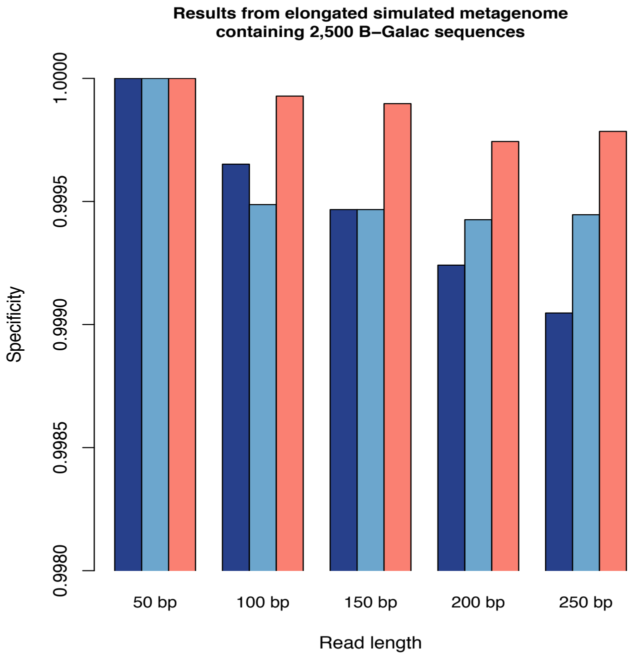

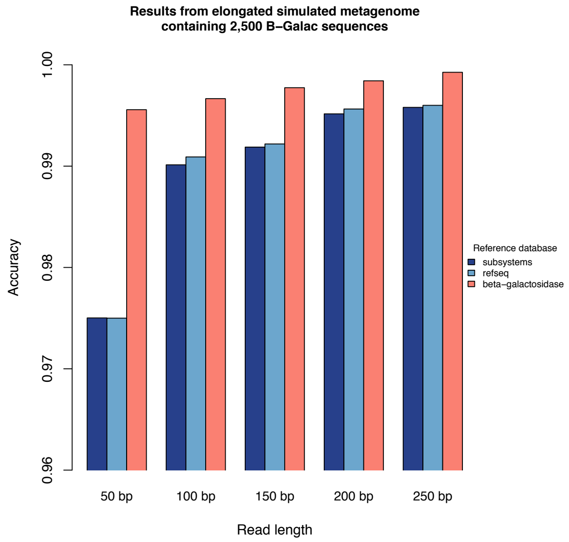

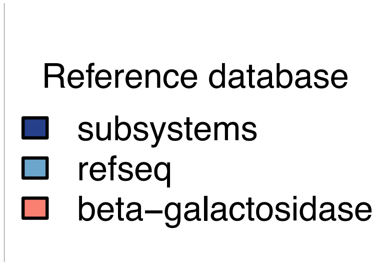

Figure S1. Sensitivity, specificity, and accuracy across different read lengths, of detecting the target beta-galactosidase sequence in simulated metagenomes using DIAMOND with “sensitive” flag and default e-value and 3 databases: NCBI RefSeq (navy), SEED Subsystems (blue), and beta-galactosidase (salmon).

Figure S2

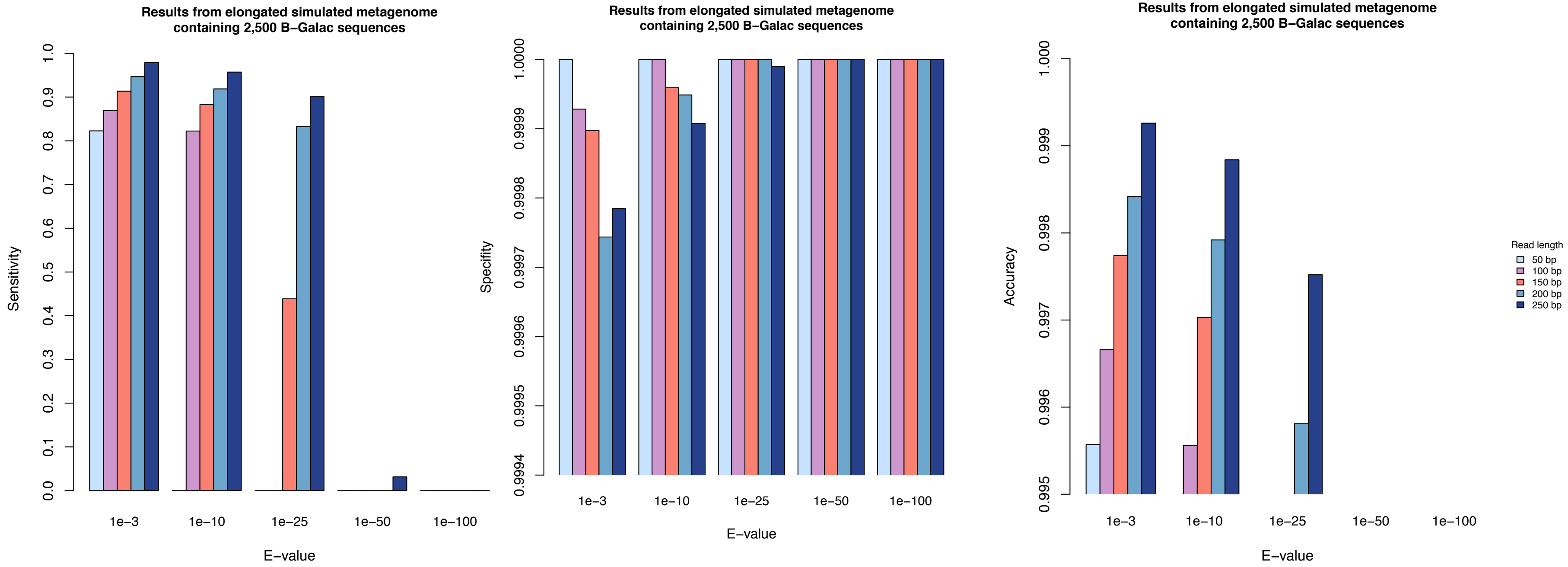

Figure S2. Sensitivity, specificity, and accuracy of detection of beta-galactosidase sequences in simulated metagenomes containing 2,500 beta-galactosidase sequences at varying e-value cutoffs and read lengths of 50 bp (light blue), 100 bp (purple), 150 bp (salmon), 200 bp (blue), and 250 bp (navy).

Figure S3

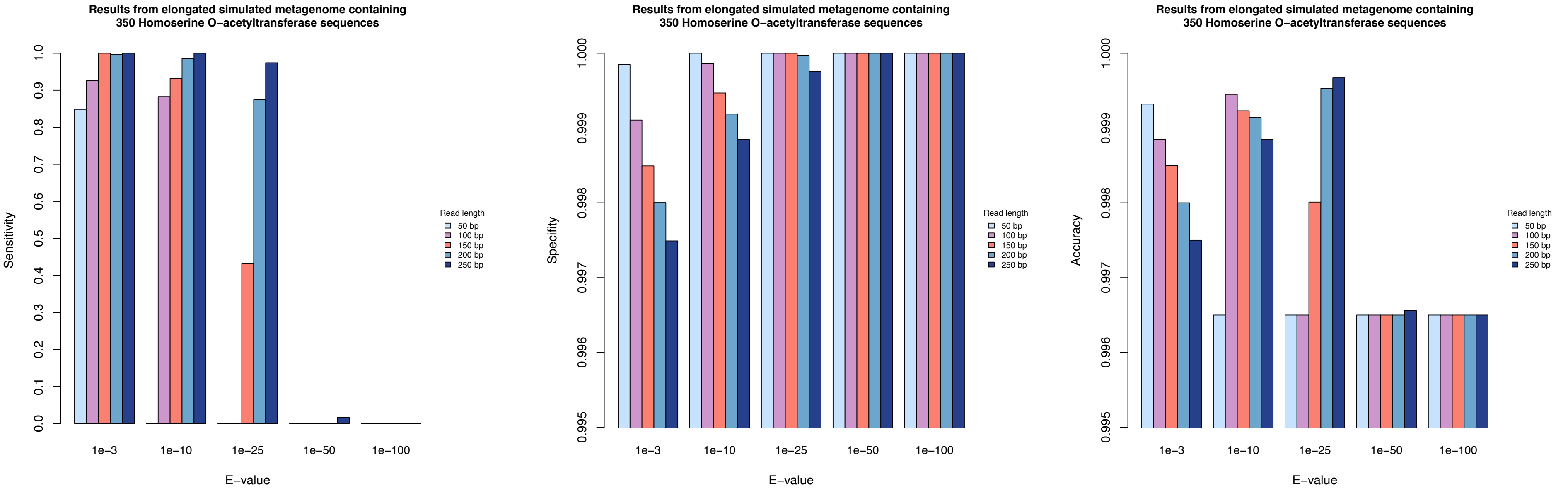

Figure S3. Sensitivity, specificity, and accuracy of Homoserine O-acetyltransferase sequence detection in simulated metagenomes containing 350 Homoserine O-acetyltransferase sequences at varying e-value cutoffs and read lengths of 50 bp (light blue), 100 bp (purple), 150 bp (salmon), 200 bp (blue), and 250 bp (navy).

Figure S4

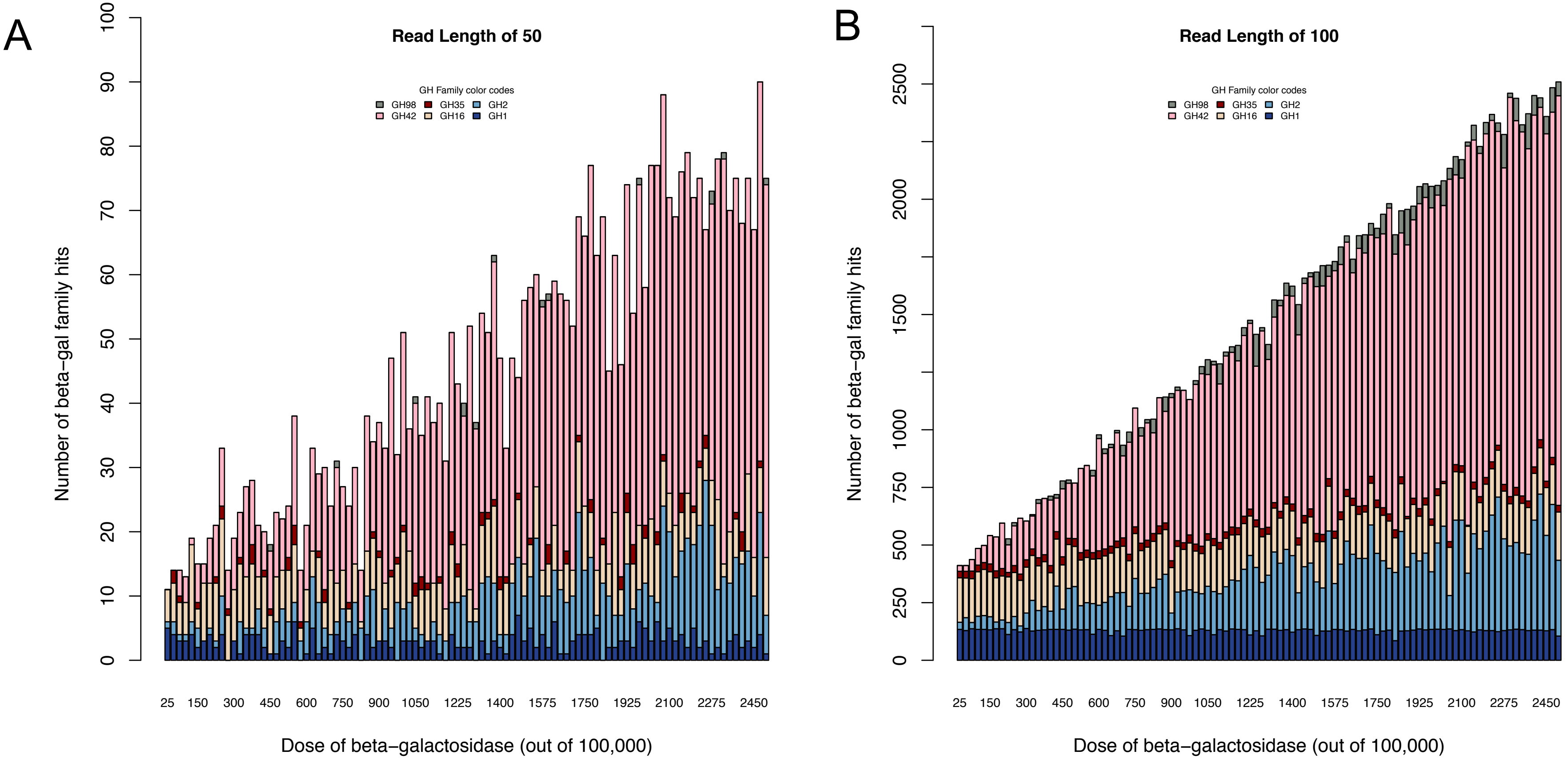

Figure S4: CAZy family distributions across varying beta-gal dosages and read lengths of 50 bp (A), 100 bp (B), 150 bp (C), 200 bp (D), and 250 bp (E). Annotations against 7 CAZy families that contain beta-galactosidase were included: GH1(navy), GH2 (blue), GH16 (tan), GH35 (red), GH42 (pink), GH59 (light green only shown in fig 3D-E), and GH98 (grey). andBased on fig 2A, a read length below 100 bp is inadequate for functional annotation. Generally as read length increases, the overall number annotations to CAZy beta-galactosidase..

Figure S4 (continued)

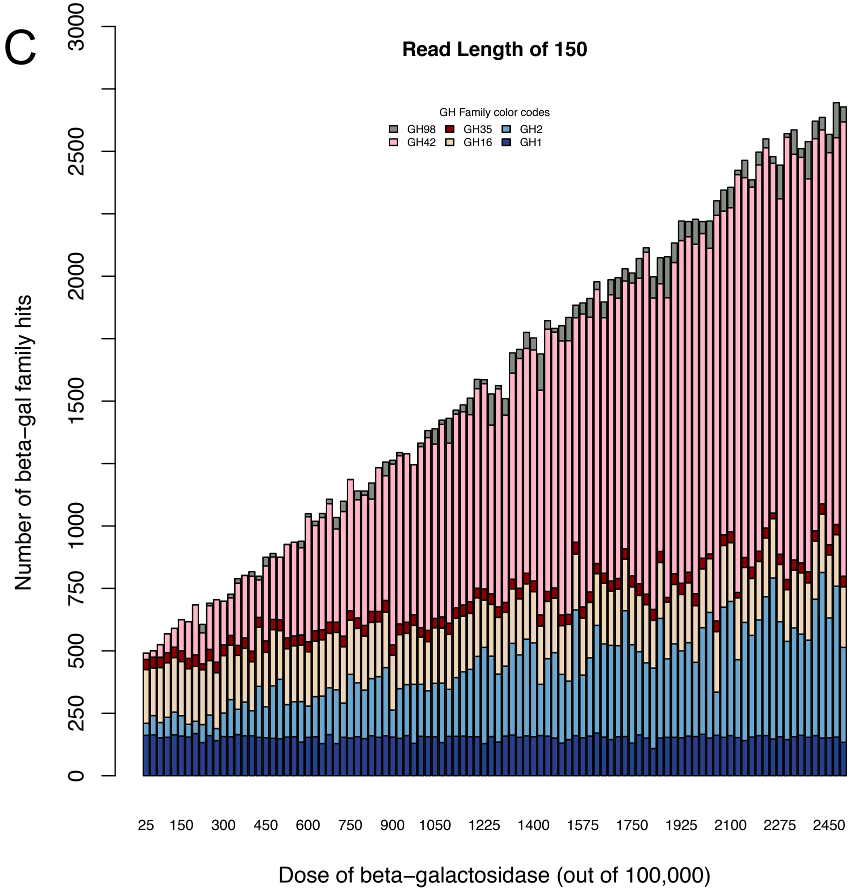

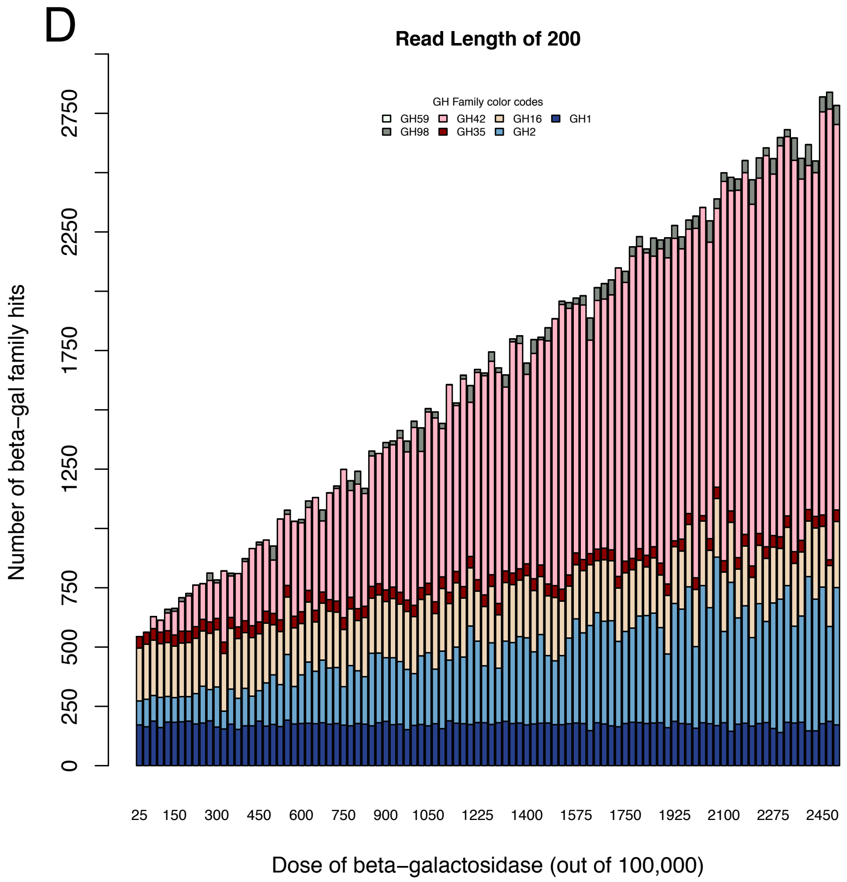

families increases. However, Glycoside Hydrolase Family 1 shows an insignificant increase in annotations with increasing dosages, likely because results include only entire CAZy families, not individual proteins, and matched proteins that are not beta-galactosidase, but in the same family

Figure S4 (continued)

E

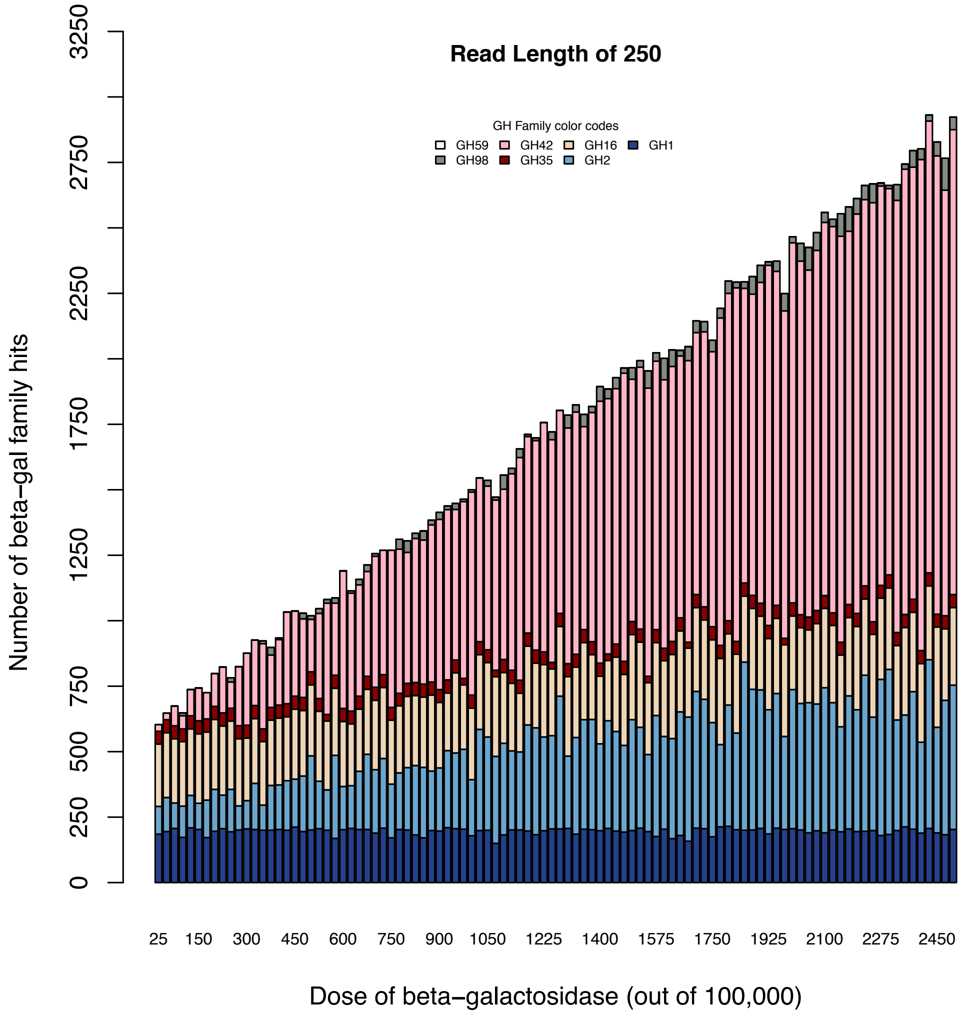

Figure S5

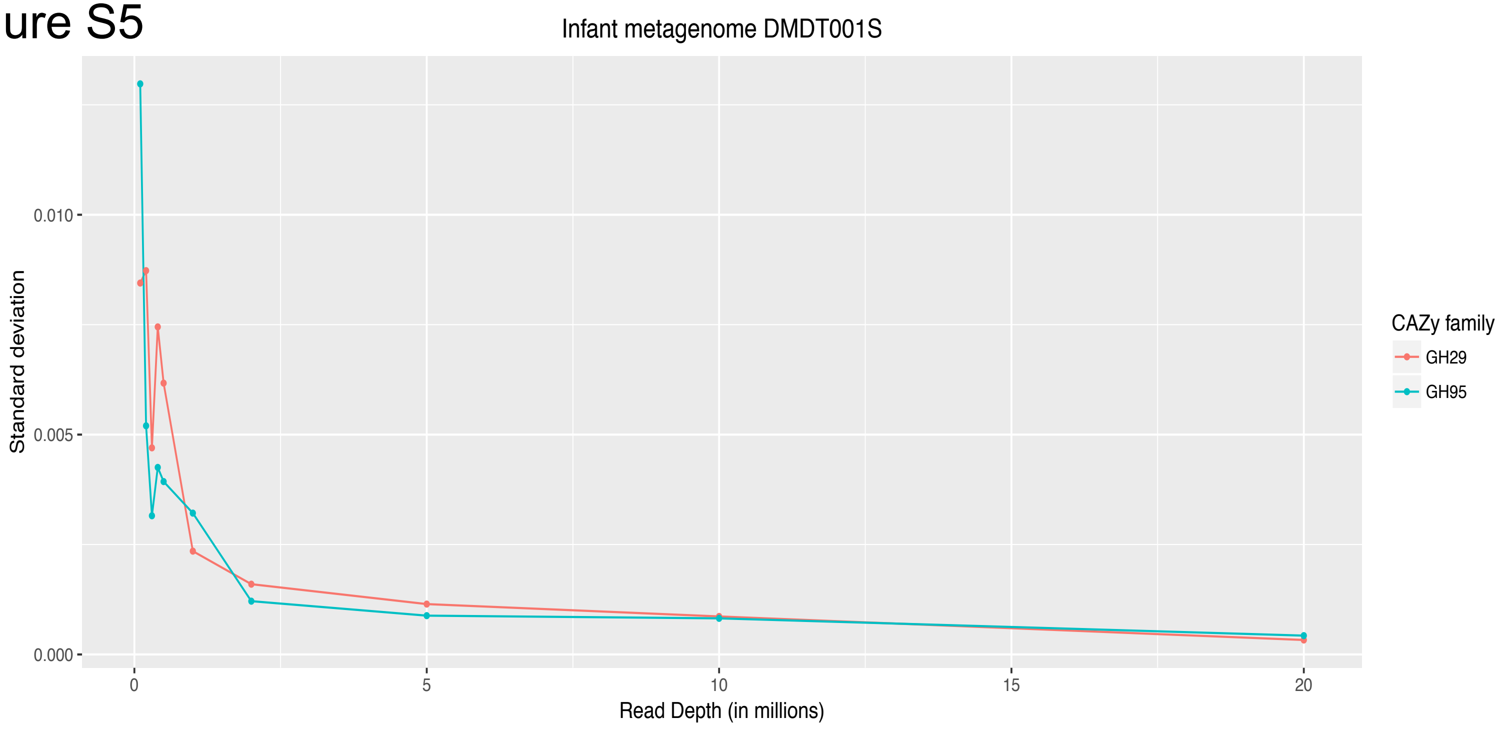

Figure S5: Standard deviation of 10 subsampled metagenomes' annotations against two fucosidase families of the CAZy database for each read depth from an infant metagenome of 30 million merged reads.
